# MYC Expression in Concert with BCL2 and BCL6 Expression Predicts Outcome in Chinese Patients with Diffuse Large B-Cell Lymphoma, Not Otherwise Specified

**DOI:** 10.1371/journal.pone.0104068

**Published:** 2014-08-04

**Authors:** Li-Xu Yan, Yan-Hui Liu, Dong-Lan Luo, Fen Zhang, Yu Cheng, Xin-Lan Luo, Jie Xu, Jie Cheng, Heng-Guo Zhuang

**Affiliations:** Department of Pathology, Guangdong General Hospital, Guangdong Academy of Medical Science, Guangzhou, China; University of Nebraska - Lincoln, United States of America

## Abstract

Recent studies provide convincing evidence that a combined immunohistochemical or fluorescence *in situ* hybridization (FISH) score of MYC, BCL2, BCL6 proteins and *MYC* translocations predicted outcome in diffuse large B-cell lymphoma (DLBCL) patients treated with rituximab, cyclophosphamide, doxorubicin, vincristine, and prednisone (R-CHOP). However, by far, all these researches are based on Western populations. Therefore, we investigate the prognostic relevance of MYC-, BCL2- and BCL6-rearrangements and protein expression by immunohistochemistry and FISH from 336 *de novo* DLBCL, NOS treated with CHOP or R-CHOP. Breaks in *MYC* and *BCL6*, and fusion in *IGH/BCL2* were detected in 9.7%, 20.0%, and 11.1% of the cases, respectively, and were not significantly associated with clinical outcomes. Protein overexpression of MYC (≥40%), BCL2 (≥70%) and BCL6 (≥50%) was encountered in 51%, 51% and 36% of the tumors, respectively. On the basis of MYC, BCL2 and BCL6 expression, double-hit scores (DHSs) and triple-hit score (THS) were assigned to all patients with DLBCL. Patients with high MYC/BCL2 DHS, high MYC/BCL6 DHS and high THS had multiple adverse prognostic factors including high LDH level, poor performance status, advanced clinical stage, high International Prognostic Index (IPI) score, and non-germinal center B-cell. In univariate analysis, high MYC/BCL2 DHS, high MYC/BCL6 DHS and high THS were associated with inferior OS and PFS in both CHOP and R-CHOP cohorts (*P*<0.05). The highly significant correlations with OS and PFS were maintained in multivariate models that controlled for IPI (*P*<0.05). DLBCLs with high DHSs and high THS share the clinical features and poor prognosis of double-hit lymphoma (*P*>0.05). These data together suggest that the immunohistochemical DHSs and THS defined a large subset of DLBCLs with double-hit biology and was strongly associated with poor outcome in patients treated with R-CHOP or CHOP.

## Introduction

Diffuse large B-cell lymphoma (DLBCL) exhibits various morphologies, immunophenotypes, genetic aberrations, and clinical courses. These features vary across geographic regions, suggesting geographic heterogeneity as a characteristic of this type of lymphoma. DLBCL constitutes 31–34% of all non-Hodgkin lymphomas in Western countries, more than 40% in Asian countries and 45.8% in china [1], [2].

The International Prognostic Index (IPI) has been confirmed to be a valid prognosticator for patients receiving standard chemotherapy [3]. However, there are considerable differences in outcome within each of risk groups, suggesting underlying biologic differences that are not encompassed by the IPI factor [4]. In addition, gene expression profiling has stratified DLBCL into prognostically different molecular subtypes based on cell of origin, including germinal center B-cell (GCB)-like, activated B-cell-like subtypes, and unclassified DLBCL [5], [6]. However, these subtypes do not reliably predict the prognosis of individual patients [7]. Furthermore, gene expression profiling is not available in most clinical laboratories. An immunophenotypical subdivision of DLBCL, not otherwise specified (NOS) into GCB and non-germinal center B-cell (non-GCB) subgroups has been proposed as prognosis predictor by different groups [8]. However, in some studies this immunophenotypic subdivision do not correlate with prognosis [9], [10], and does not currently determine therapy [11].

Recent studies provide convincing evidence that a DLBCL population characterized by the coexpression of MYC and BCL2 proteins by IHC has a poor prognosis with standard rituximab, cyclophosphamide, doxorubicin, vincristine, and prednisone (R-CHOP) immunochemotherapy [12]–[14]. More recently, Heike et al. [15] have reported that a combined immunohistochemical or fluorescence *in situ* hybridization (FISH)/immunohistochemical score, including MYC, BCL2, BCL6 protein expressions and *MYC* translocations, predicts outcome in DLBCL patients independent of the IPI following treatment with R-CHOP.

DLBCL in China appears to have many characteristics different from those in Western countries; however, by far, all these researches are based on Western populations. In this study, therefore, we aimed to comprehensively assess the prognostic impact of protein expression patterns of MYC, BCL2, and BCL6 in concert with the chromosomal translocations targeting *MYC*, *BCL2*, and *BCL6* in a Chinese cohort of 336 *de novo* DLBCL, NOS patients treated with CHOP or R-CHOP.

## Materials and Methods

### Patient Selection

We studied 336 cases of *de novo* DLBCL, NOS from patients who were treated with 6 or 8 cycles of CHOP treatment with or without 8 applications of rituximab. Patients were selected based on the availability of baseline clinical and outcome data, and sufficient formalin-fixed paraffin-embedded (FFPE) tissue from the pre-treatment biopsy sample for representation in tissue microarrays (TMAs). The archived FFPE tissues were obtained from the Department of Pathology, Guangdong General Hospital between January 2000 and October 2012. A consensus diagnosis of DLBCL was confirmed by two expert pathologists according to 2008 World Health Organization (WHO) classification criteria [11]. Median follow-up time was 37 months (range, 1 to 145 months). The Research Ethics Committee of Guangdong General Hospital & Guangdong Academy of Medical Science reviewed and approved the study (No. GDREC2013122H) according to the principles expressed in the Declaration of Helsinki. The Research Ethics Committee specifically waived the need for informed consent for this project.

### TMA Construction and Immunohistochemistry (IHC)

TMAs that contained three representative 2.0-mm cores from each tumor of the cases were prepared with a tissue microarrayer (Beecher Instruments, Silver Spring, MD). Immunohistochemical stainings were performed using Real Envision Kit (K5007, DAKO, Carpinteria, CA, USA) on two automated immunostaining instruments (Discovery XT, Ventana Medical Systems, Tucson, AZ, USA; Leica Bond-Max, Leica Biosystems, Germany) according to the manufacturer’s instructions. Internal control cores were present in each TMA. Sections were subjected to staining protocols with the following antibodies: MYC (clone Y69; Epitomics, Burlingame, CA, USA), BCL2 (clone 124; DAKO, Glostrup, Denmark), BCL6 (clone PG-B6p; DAKO), and Ki67 (clone MIB1; DAKO; **Table S1**). All cases were scored semiquantitatively in 10% increments as previously reported [16] by two observers without knowledge of patient outcome or FISH results. Discrepant scoring of >10% was resolved using a multiheaded microscope to reach a consensus score.

Data were analyzed using MedCalc statistical software to determine the optimal survival cut-off points for dichotomizing expression of MYC protein (≥40%), BCL2 protein (≥70%), BCL6 protein (≥50%) and Ki67 index (≥90%). These cut points correspond to the maximum Chi-Square value of the Kaplan-Meier test for overall survival (OS) between groups above and below the cut-point threshold.

### FISH

Interphase FISH was performed on TMAs of 150 cases from the same cohort as previously described [17]. The Vysis LSI *MYC* dual color, break apart rearrangement probe, the Vysis LSI *BCL6* dual color, break apart rearrangement probe and the Vysis LSI *IGH/BCL2* dual fusion translocation probe (Abbott Molecular, Abbott Park, IL) were used. FISH signals were analyzed using a fluorescence microscope (Olympus BX51, Tokyo, Japan) equipped with a DP72 camera and DP2-BSW software (Olympus, Tokyo, Japan). Patient cases with break-apart signals in >10% of nuclei were considered positive for the presence of a translocation. The signal distribution was evaluated by two independent observers (Dong-Lan Luo and Jie Cheng). In case of discordant results between the two observers, a third investigator (Jie Xu) was involved.

### Statistical Analysis

Statistical analysis was prepared using the Statistical Package of MedCalc statistical software (version 12.7.4; MedCalc, Mariakerke, Belgium) and Social Sciences (SPSS, version 20.0; SPSS, Chicago, IL, USA). A receiver operating characteristic curves were constructed to estimate the optimal cut-off points for of MYC, BCL2 and BCL6 proteins as the predictors for OS. Pearson’s Chi-Square test, Fisher’s exact test, Correction for continuity and Spearman rank correlation analysis were used to determine association and correlation between variables. Survival analyses were plotted using Kaplan-Meier curves and compared using the log-rank test. Univariate and Multivariate survival analyses were analyzed by Cox proportional hazards regression models. The results were considered statistically significant when two-sided *P*<0.05.

## Results

### Clinical and Immunophenotypical Characteristics

**Figure 1:**
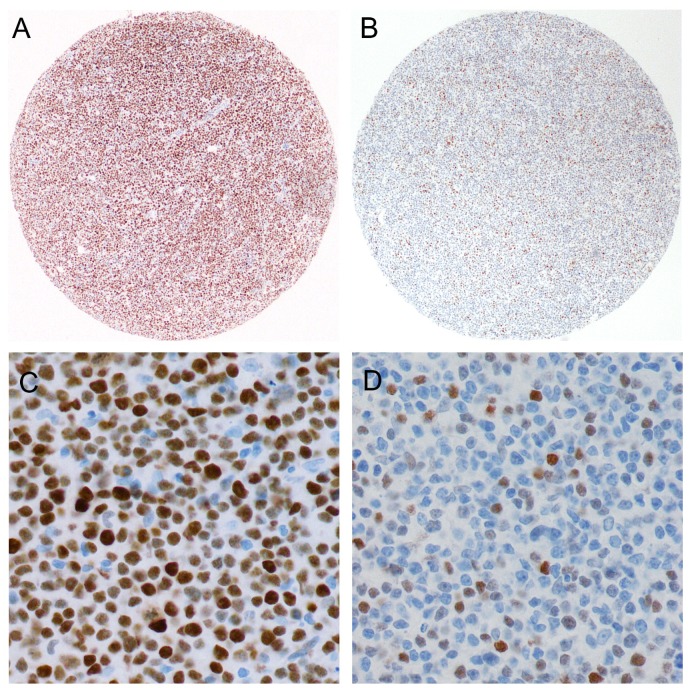
Tissue microarray based representative immunohistochemical analysis of MYC protein expression in DLBCL. The MYC staining pattern is distinctly nuclear. (A, C) DLBCL scored as having ≥40% MYC-positive lymphoma cells. (B, D) DLBCL scored as having <40% MYC-positive lymphoma cells. A and B original magnification, ×40. C and D original magnification, ×400.

We studied the series of 336 DLBCL, NOS tumor samples by IHC on the TMAs using antibodies for MYC, BCL2, BCL6 and Ki67 (**Figure 1**). In addition, ten patient cases which could not be scored for technical reasons were also studied on the corresponding whole tissue sections from the original FFPE tumor blocks. Stainings of the four markers were reliably interpretable in all the 336 samples. The clinical and immunophenotypical characteristics of the DLBCL, NOS are provided in **Table 1**. DLBCL, NOS was immunophenotypically subdivided into CD5-positive, GCB and non-GCB subgroups according to the Hans classifier [8] and the 2008 WHO classification [11]. Fifteen DLBCL, NOS (4%) were of CD5-positive-subgroup, 90 (27%) of GCB-subgroup, and 231 (69%) of non-GCB-subgroup.

**Table 1:** Clinical and immunophenotypical characteristics of DLBCL, NOS patients.

	All patients (N = 336)			Patients with FISH data (n = 150)		
Characteristic	No.		%	No.	%	*P*
**Male**	195/336		58	86/150	57	0.885
**Age, years median (range)**	57 (7,87)			58 (7,86)		
**LDH>upper limit of normal**	129/336	38		59/150	39	0.844
**ECOG PS ≥2** #	51/336	15		29/150	19	0.254
**Ann Arbor stage III/IV**	156/336	46		68/150	45	0.823
**Extranodal sites ≥2**	57/336	17		22/150	15	0.526
**IPI score** $						
0 or 1	170/336	51		75/150	50	0.904
2	83/336	25		42/150	28	0.442
3	59/336	18		21/150	14	0.328
4 or 5	24/336	7		12/150	8	0.739
**Extranodal involvement**	235/336	70		100/150	67	0.471
**Bone marrow involvement**	36/336	11		12/150	8	0.354
**Immunohistochemical subgroups**						
CD5-positive DLBCL	15/336	4		11/150	7	0.194
GCB	90/336	27		47/150	31	0.303
non-GCB	231/336	69		92/150	61	0.110
**High MYC expression**	170/336	51		88/150	59	0.100
**High BCL2 expression**	171/336	51		84/150	56	0.298
**High BCL6 expression**	121/336	36		65/150	43	0.125
**High Ki67 expression**	176/336	52		92/150	61	0.067
**R-CHOP**	125/336	37		64/150	43	0.254
**CHOP**	211/336	63		86/150	57	0.254
**Median follow-up time, months**	37				27	
**5-year OS**	66				70	
**5-year PFS**	47				40	

*P* values were derived from Pearson’s Chi-Square test. ECOG PS, Eastern Cooperative Oncology Group performance status.

# ECOG PS ranges from 0 to 4, where higher score indicates greater degree of impairment.

$ IPI score ranges from 0 to 5, with 0 indicating absence of prognostic factors and 5 indicating presence of all prognostic factors.

### High Double-Hit Scores (DHSs) and Triple-Hit Score (THS) are Associated With High-Risk Clinicopathologic Parameters

One hundred and seventy tumors (51%) showed high MYC expression, 171 (51%) showed high BCL2 expression and 121 (36%) showed high BCL6 expression (**Table 1**). MYC overexpression was associated with high lactate dehydrogenase (LDH) level (*P* = 0.002) and high IPI score (*P* = 0.043; **Table 2**). BCL2 overexpression was associated with poor performance status (*P* = 0.014; **Table 2**). However, no significant differences were observed with regard to clinical characteristics among low and high BCL6 groups.

**Table 2:** Patient clinical and immunophenotypical characteristics of patients with DLBCL, NOS in relation to protein expressions.

		MYC			BCL2			BCL6		
Characteristic	All patients (n = 336)	Low (n = 166)	High (n = 170)	*P*	Low (n = 165)	High (n = 171)	*P*	Low (n = 215)	High (n = 121)	*P*
	N (%)	N (%)	N (%)		N (%)	N (%)		N (%)	N (%)	
LDH>ULN	129 (38)	50 (30)	79 (46)	**0.002**	58 (35)	71 (42)	0.230	84 (39)	45 (37)	0.734
ECOG PS ≥2	51 (15)	19 (11)	32 (19)	0.060	17 (10)	34 (20)	**0.014**	34 (16)	17 (14)	0.665
Stage III/IV	156 (46)	70 (42)	86 (51)	0.122	75 (45)	81 (47)	0.725	108 (50)	48 (40)	0.062
Extranodal sites ≥2	57 (17)	29 (17)	28 (16)	0.807	27 (16)	30 (18)	0.773	39 (18)	18 (15)	0.444
IPI score of 3–5	83 (25)	33 (20)	50 (29)	**0.043**	36 (20)	51 (29)	0.056	59 (27)	24 (20)	0.121
Immunohistochemical subgroups										
CD5-positive	15 (4)	6 (4)	9(6)	0.658*	5 (2)	10 (7)	**0.001** *	12 (6)	3 (3)	**0.004** *
GCB	90 (27)	47 (28)	43 (25)	0.595#	59 (36)	31 (18)	**<0.001** #	45(21)	45 (37)	**0.002** #
non-GCB	231 (69)	113 (68)	118 (69)		101 (62)	130 (75)		158 (73)	73 (60)	

*P* values were derived from Pearson’s Chi-Square test. Bold font indicates significance. ULN, upper limit of normal.

*P value CD5+ *vs.* GCB *vs.* non-GCB.

# *P* value GCB *vs.* non-GCB.

Using the optimal survival cut-off points as described in Methods section, we assigned each patient a THS that ranged from 0 to 2. Each patient was given one point for each of the two markers (MYC and BCL2) expressed at or above the cut-off points, and one point for BCL6 expressed below the cut-off point. The DHSs of MYC/BCL2, MYC/BCL6 and BCL2/BCL6 were calculated as described previously [16]. Patients with high MYC/BCL2 DHS had multiple adverse clinical factors including high LDH level (*P* = 0.007), poor performance status (*P* = 0.010), and high IPI score (*P* = 0.038; **Table 3**). Similarly, patients with high MYC/BCL2 DHS was associated with high LDH level, high clinical stage, and high IPI score (*P*>0.05). However, no significant differences were observed with regard to clinical factors included in **Table 3** among BCL2/BCL6 DHS 0, 1 and 2. Notably, patients with high THS had multiple adverse prognostic factors including high LDH level (*P* = 0.024), poor performance status (*P* = 0.025), high clinical stage (*P* = 0.008), and high IPI score (*P* = 0.009; **Table 3**).

**Table 3:** Patient clinical and immunophenotypical characteristics of patients with DLBCL, NOS in relation to DHS and THS.

		MYC/BCL2				MYC/BCL6				BCL2/BCL6				MYC/BCL2/BCL6				
Characteristic	All patients(n = 336)	DHS 0(n = 90)	DHS 1(n = 151)	DHS 2(n = 95)	*P*	DHS 0(n = 57)	DHS 1(n = 173)	DHS 2(n = 106)	*P*	DHS 0(n = 65)	DHS 1(n = 156)	DHS 2(n = 115)	*P*	THS 0(n = 27)	THS 1(n = 131)	THS 2(n = 109)	THS 3(n = 69)	*P*
	N (%)	N (%)	N (%)	N (%)		N (%)	N (%)	N (%)		N (%)	N (%)	N (%)		N (%)	N (%)	N (%)	N (%)	
LDH>ULN	129 (38)	28 (31)	52 (34)	49 (52)	**0.007**	17 (30)	61 (35)	51 (48)	**0.035**	23 (35)	57 (37)	49 (43)	0.512	7 (26)	47 (36)	38 (35)	37 (54)	**0.024**
ECOG PS ≥2	51 (15)	8 (9)	20 (13)	23 (24)	**0.010**	6 (11)	24 (14)	21 (20)	0.228	4 (6)	26 (17)	21 (18)	0.073	2 (7)	12 (9)	23 (21)	14 (20)	**0.025**
Stage III/IV	156 (46)	36 (40)	73 (48)	47 (49)	0.355	19 (33)	80 (46)	57 (54)	**0.044**	28 (43)	67 (43)	61 (53)	0.215	12 (44)	47 (36)	63 (58)	34 (49)	**0.008**
Extranodal sites ≥2	57(17)	13 (14)	30 (20)	14 (15)	0.440	8 (14)	31 (18)	18 (17)	0.795	11 (17)	23 (15)	23 (20)	0.522	4 (15)	20 (15)	22 (20)	11 (16)	0.751
IPI score of 3–5	83 (25)	14 (16)	39 (26)	30 (32)	**0.038**	7 (12)	43 (25)	33 (31)	**0.029**	11 (17)	36 (23)	36 (31)	0.081	3 (11)	23 (18)	36 (33)	21 (30)	**0.009**
Immunohistochemical subgroups																		
CD5-positive	15 (4)	2 (2)	7 (5)	6 (6)	NS*	3 (5)	3 (2)	9 (8)	NS*	0 (0)	8 (5)	7 (6)	NS*	0 (0)	5 (4)	4 (4)	6 (9)	NS*
GCB	90 (27)	29 (32)	48 (32)	13 (14)	**0.004** #	21 (37)	50 (29)	19 (18)	**0.034** #	33 (51)	38 (24)	19 (17)	**<0.001** #	12 (44)	47 (36)	21 (19)	10 (14)	**0.001** #
non-GCB	231 (69)	59 (66)	96 (63)	76 (80)		33 (58)	120 (69)	78 (74)		32 (49)	110 (71)	89 (77)		15 (56)	79 (60)	84 (77)	53 (77)	

*P* values were derived using Pearson’s Chi-Square test. Bold font indicates significance.

*NS, not suitable for chi-square test among CD5+ *vs.* GCB *vs.* non-GCB, because more than 1/5 of the expected values were less than five.

# *P* value GCB *vs.* non-GCB.

One hundred and seventy-five tumors (52.1%) showed high Ki67 expression. No significant differences were observed with regard to LDH level, performance status, clinical stage, extranodal sites, IPI score, or immunohistochemical subgroups (GCB *vs*. non-GCB) among high Ki67 proliferation index and low Ki67 proliferation index groups.

### High DHS and THS Show Non-GCB Predominance

The immunophenotypical characteristics of patients with DLBCL, NOS in relation to protein expressions are shown in **Table 2**. MYC expression demonstrated no correlation with immunohistochemical subgroups of DLBCL. Considering BCL2 and BCL6 expression individually, the high BCL2 expression group had a significantly higher frequency of non-GCB than the low BCL2 group (75% *vs.* 62%, *P*<0.001). However, low BCL6 expression group had a higher frequency of non-GCB than the high BCL6 group (73% *vs.* 60%, *P* = 0.002).

MYC/BCL2 coexpression (DHS 2) correlated significantly with the non-GCB immunohistochemical subgroup (*P* = 0.004; **Table 3**). Of total 336 cases of DLBCL, NOS, with MYC/BCL2 DHS 2, 76 (80%) were of the non-GCB-DLBCL. By contrast, only 96 (63%) of DLBCL with MYC/BCL2 DHS 1 were of the non-GCB-DLBCL, and 59 (66%) of DLBCL with MYC/BCL2 DHS 0 were of the non-GCB-DLBCL (**Table 3**). Similar results were found for MYC/BCL6, BCL2/BCL6 and MYC/BCL2/BCL6 coexpression.

### FISH Studies and Double-Hit Lymphoma (DHL)

Initially, we created a pilot series of 336 DLBCL, NOS tumor samples spotted on TMAs. After IHC analysis, sufficient materials of 150 cases on the TMAs were available for complete FISH analysis. The clinical and immunophenotypical characteristics of the 150 patients are shown in **Table 1**. Of 150 DLBCL specimens hybridized, 144 (96%), 140 (93%) and 135 (90%) samples were successfully interpretable for the *MYC* and *BCL6* break-apart probes and the *IGH/BCL2* fusion probe used, respectively. *MYC*, *BCL6*, and *BCL2* gene translocations were observed in 9.7%, 20.0%, and 11.1% of the cases, respectively (**Figure S1**).

No significant corrections were observed between *MYC* and *BCL6* gene breaks and clinical characteristics, including LDH level, performance status, clinical stage, extranodal sites and IPI. Patients with high IPI score had higher *IGH/BCL2* fusion rate than those with low IPI score (28.6% *vs*. 7.4%; *χ* ^2^ test, correction for continuity, *P* = 0.006). No correlations between gene translocations and immunohistochemical subgroups were seen (**Table S2**). The lack of significant differences in gene translocations between the GCB and the non-GCB DLBCL subgroups indicates that abnormalities of *MYC, BCL6 and BCL2* may be a more global phenomenon in Chinese DLBCL and not restricted to particular immunohistochemical subgroups. Breaks in *MYC* and *BCL6*, as well as fusion in *IGH/BCL2* did not predict OS and progression-free survival (PFS) in univariate and multivariate analyses in rituximab treated patients. We observed similar results for patients treated without rituximab.

We further investigated the double-hit lymphoma (DHL) in our series. One of 134 DLBCL (0.7%) had concurrent translocation of *MYC* and *BCL2*, 1/140 (0.7%) of *MYC* and *BCL6*, and 4/131 (3.1%) of *BCL2* and *BCL6.* Thus those were determined to have DHL. No triple-hit lymphoma was detected. Given those low incidences, DHL data sets were pooled for subsequent analyses. These patients with DHL (by FISH) appeared to have more adverse clinical risk factors, including higher levels of LDH, worse performance status, and higher IPI scores than the patients without DHL (non-DHL) (*P*>0.05; **Table S3**). The lack of significance is probably due to low statistical power from small group sizes. However, BCL2 protein was expressed in a higher proportion of the DHL patients than the non-DHL patients with THS 0/1 (*P* = 0.005, **Table S3**). Three of 6 DHL patients were treated with R-CHOP. The 5-year OS rate of patients with DHL treated with R-CHOP or CHOP in this study (50%) was lower than that of the non-DHL patients (70%), although *P*>0.05 (**Figure S2**). Six but too few patient cases with DHL could not preclude any meaningful conclusions. However, the 5-year OS of the DHL patients was poor compared with outcome among non-DHL patients with THS 0/1 (50% *vs*. 83%; *P* = 0.048; **Figure S2**). When comparing the patients with DHL with non-DHL patients in the THS-2/3 group, no significant differences were found between clinical characteristics or survival, implying that patients with DHL and those non-DHL patients in the THS-2/3 group are clinically similar and indicating that they share the same unfavorable double-hit tumor biology (**Table S3; Figure S2**).

### Protein Expressions Predict Corresponding Gene Translocations

To analyze the diagnostic performance of MYC, BCL2 and BCL6 protein expressions for corresponding gene translocations with the highest specificity and sensitivity, we used ROC curve analysis to determine the optimal cut-off values of the percentages of protein positive cells. The optimal cut-off values for MYC, BCL2 and BCL6 were ≥90%, ≥70% (equal to predetermined threshold) and ≥20%. The results signify that immunostaining for MYC, BCL2 and BCL6 appears to be an excellent test with high specificity for the presence of *MYC* breaks, *IGH/BCL2* fusion and *BCL6* breaks as detected by FISH (**Table S4**). High MYC expression showed correlation with *MYC* breaks in DLBCL, NOS (Spearman rank correlation analysis, Spearman’s rho = 0.481, *P*<0.001), the GCB subgroup (Spearman’s rho = 0.623, *P*<0.001) and the non-GCB subgroup (Spearman’s rho = 0.556, *P*<0.001). High BCL6 expression showed correlation with *BCL6* breaks in DLBCL, NOS (Spearman’s rho = 0.223, *P* = 0.008) and the non-GCB subgroup (Spearman’s rho = 0.277, *P* = 0.011) but showed no significant correlation in the GCB subgroup. Inversely, high BCL2 expression showed correlation with *IGH/BCL2* fusion in and the GCB subgroup (Spearman’s rho = 0.369, *P* = 0.015) but showed no significant correlation in DLBCL, NOS or the non-GCB subgroup. Chi-Square test for gene translocation and protein expression in are shown in **Table S5–S7**.

### High DHSs and THS Predict Poor Prognosis in DLBCL, NOS

**Figure 2:**
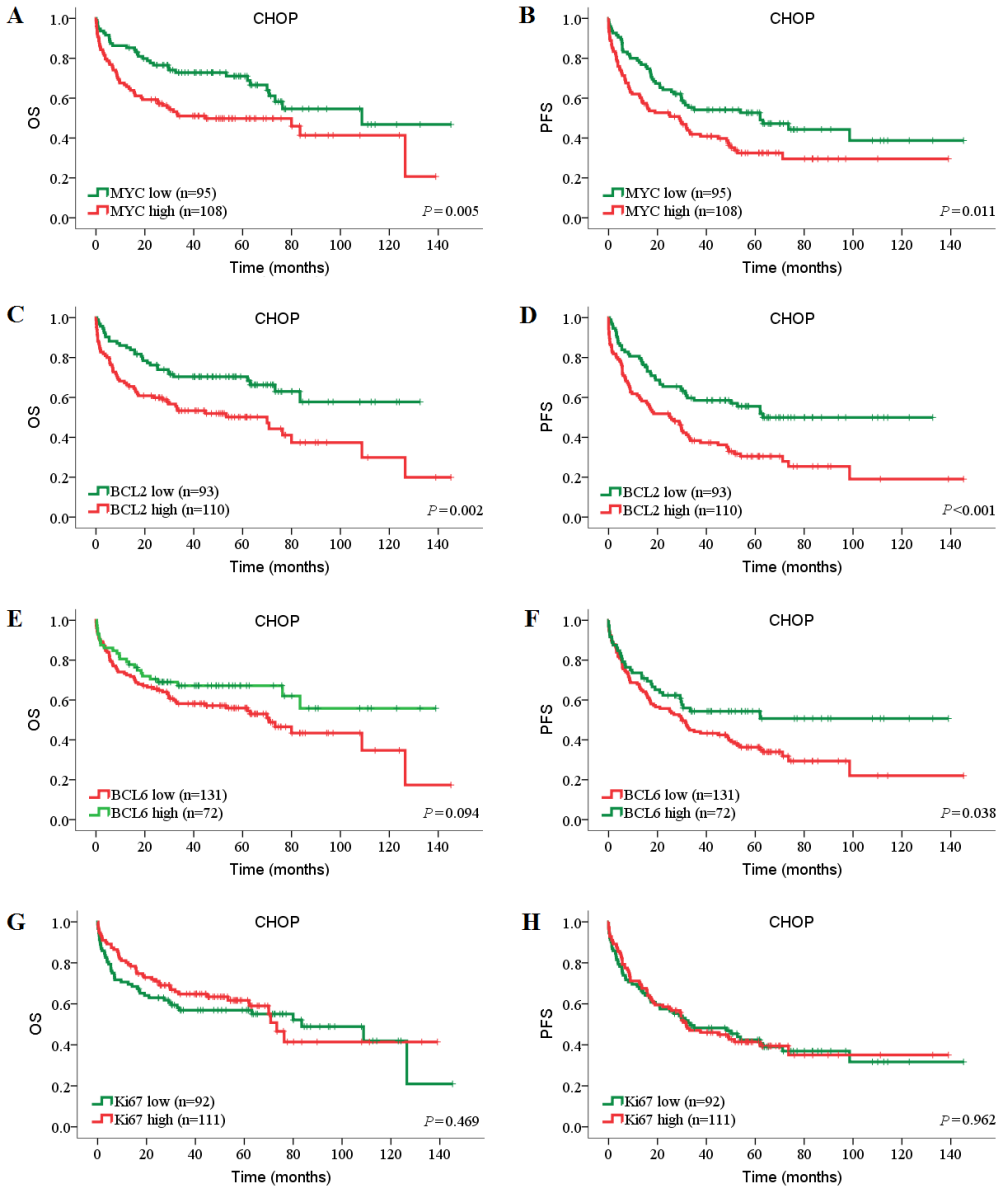
Prognostic impact of MYC, BCL2, BCL6 and Ki67 expression in DLBCL patients treated with CHOP. Patients’ tumors were stained for (A, B) MYC, (C, D) BCL2, (E, F) BCL6, and (G, H) Ki67. The numbers of patients showing negative or positive stainings (MYC≥40%, BCL2≥70%, BCL6≥50%, Ki67≥90%), cut-off values, and *P* values are indicated.

**Figure 3:**
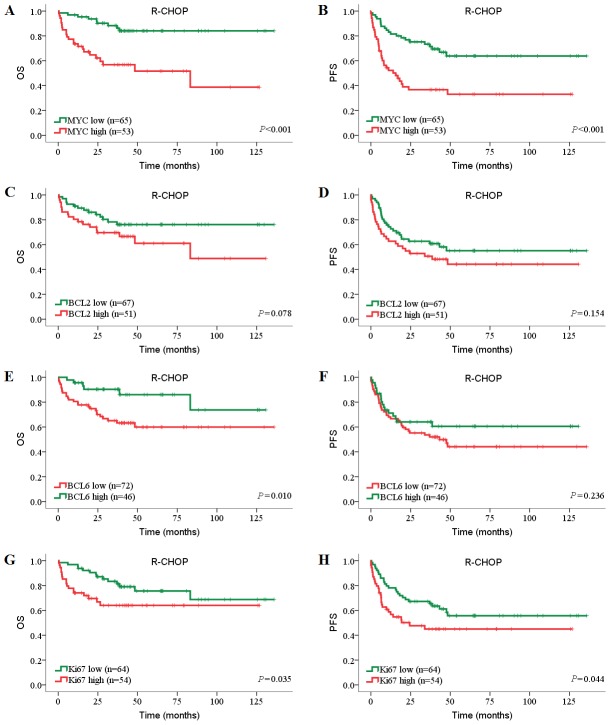
Prognostic impact of MYC, BCL2, BCL6 and Ki67 expression in DLBCL patients treated with R-CHOP. Patients’ tumors were stained for (A, B) MYC, (C, D) BCL2, (E, F) BCL6, and (G, H) Ki67. The numbers of patients showing negative or positive stainings (MYC≥40%, BCL2≥70%, BCL6≥50%, Ki67≥90%), cut-off values, and *P* values are indicated.

Because several recent studies have shown that prognostic value of biomarkers have changed significantly in rituximab era [18]–[20], we evaluated the candidate prognostic factors separately in the CHOP and R-CHOP cohorts. The survival curves showed that high MYC expression was significantly associated with inferior OS and PFS in both CHOP and R-CHOP cohorts (*P*<0.05, log-rank tests; **Figure 2A–B** **,** **Figure 3A–B**). High BCL2 expression, alone, was significantly associated with inferior OS and PFS in CHOP cohort (OS: *P* = 0.002; PFS, *P*<0.001) but not in R-CHOP cohort (**Figure 2C–D** **,** **Figure 3C–D**). However, low BCL6 expression showed limited prognostic impact on inferior outcome in both CHOP and R-CHOP cohorts (PFS of CHOP cohort: *P* = 0.038; OS of R-CHOP cohort: *P* = 0.010; **Figure 2E–F** **,** **Figure 3E–F**). Interestingly, in contrast with BCL2, Ki67 was significantly associated with inferior OS and PFS in R-CHOP cohort (OS: *P* = 0.035; PFS, *P* = 0.044) but not in CHOP cohort (**Figure 2G–H** **,** **Figure 3G–H**).

**Figure 4:**
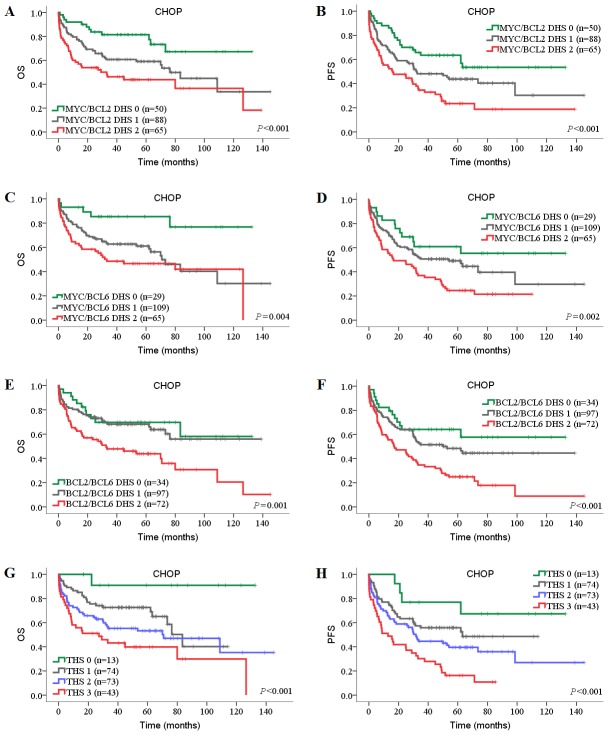
Prognostic impact of DHSs and THS in DLBCL patients treated with CHOP. (A, B) OS (A) and PFS (B) of patients with MYC/BCL2 DHS. (C, D) OS (C) and PFS (D) of patients with MYC/BCL6 DHS. (E, F) OS (E) and PFS (F) of patients with BCL2/BCL6 DHS. (G, H) OS (G) and PFS (H) of patients with MYC/BCL2/BCL6 THS. OS, overall survival; PFS, progression-free survival; DHS, double-hit score; THS, triple-hit score.

**Figure 5:**
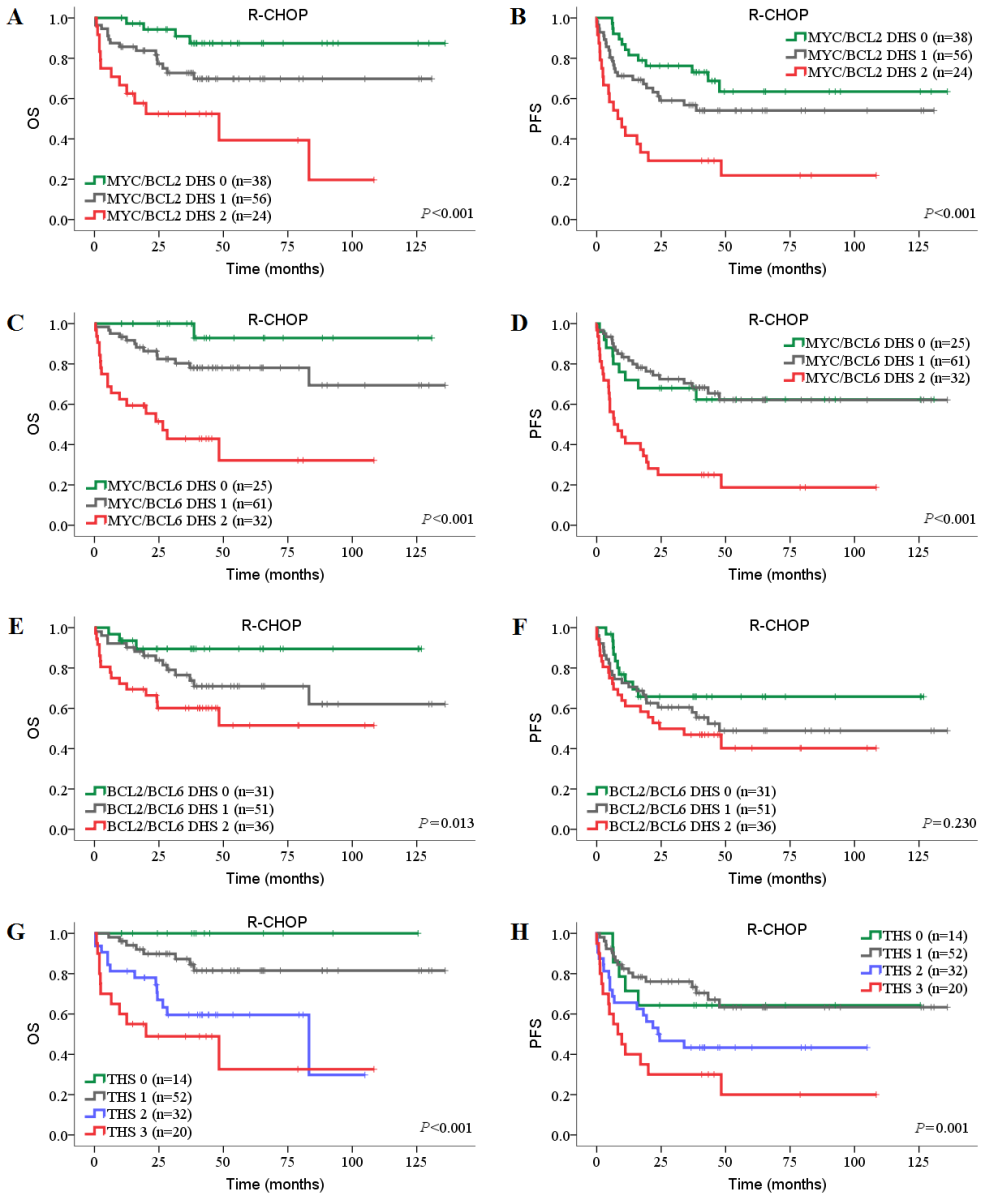
Prognostic impact of DHSs and THS in DLBCL patients treated with R-CHOP. (A, B) OS (A) and PFS (B) of patients with MYC/BCL2 DHS. (C, D) OS (C) and PFS (D) of patients with MYC/BCL6 DHS. (E, F) OS (E) and PFS (F) of patients with BCL2/BCL6 DHS. (G, H) OS (G) and PFS (H) of patients with MYC/BCL2/BCL6 THS. OS, overall survival; PFS, progression-free survival; DHS, double-hit score; THS, triple-hit score.

Next, we investigated the prognostic impact of DHSs and THS on DLBCL patients in both CHOP and R-CHOP cohorts. In univariate analysis, high MYC/BCL2 DHS, high MYC/BCL6 DHS and high THS were associated with inferior OS and PFS in both CHOP and R-CHOP cohorts (**Table 4**; **Table 5**). In the CHOP cohort, compared with individual BCL2 and BCL6 protein, the negative prognostic impact of high BCL2 protein and low BCL6 protein was amplified when BCL2/BCL6 DHS was high (**Table 4** **;** **Figure 4E–F**). In the R-CHOP cohort, high BCL2 protein expression was only associated with inferior OS and PFS when MYC protein was coexpressed (*P*<0.001; **Table 5**, **Figure 5A–B**). BCL2/BCL6 DHS showed limited prognostic impact in R-CHOP cohort (univariate model: OS, *P* = 0.005, PFS, *P* = 0.089; multivariate model: OS, *P* = 0.006, PFS, *P* = 0.132).

**Table 4:** Univariate Cox models for DLBCL, NOS patients treated with CHOP.

		OS			PFS		
Characteristic	n#	HR	95% CI	*P*	HR	95% CI	*P*
MYC protein low *vs*. High	203	1.83	1.19–2.82	**0.006**	1.60	1.11–2.31	**0.012**
BCL2 protein low *vs*. High	203	1.95	1.26–3.01	**0.003**	1.94	1.34–2.82	**0.001**
BCL6 protein low *vs*. High	203	0.68	0.43–1.07	0.096	0.66	0.44–0.98	**0.040**
High Ki67 protein low *vs*. High	203	0.86	0.56–1.30	0.470	0.99	0.69–1.42	0.962
MYC/BCL2 DHS 0 *vs*. 1 *vs*. 2	203	1.79	1.33–2.40	**<0.001**	1.67	1.30–2.14	**<0.001**
MYC/BCL6 DHS 0 *vs*. 1 *vs*. 2	203	1.71	1.24–2.37	**0.001**	1.63	1.23–2.16	**0.001**
BCL2/BCL6 DHS 0 *vs*. 1 *vs*. 2	203	1.70	1.24–2.34	**0.001**	1.73	1.32–2.28	**<0.001**
THS 0 *vs*. 1 *vs*. 2 *vs*. 3	203	1.70	1.33–2.18	**<0.001**	1.65	1.33–2.04	**<0.001**
GCB *vs*. Non-GCB	203	1.56	0.95–2.56	0.082	1.75	1.12–2.72	0.013
IPI score of 0–2 *vs.* 3–5	203	2.87	1.89–4.36	**<0.001**	2.21	1.52–3.19	**<0.001**
MYC break negative *vs*. positive	76	1.22	0.37–4.04	0.745	1.28	0.50–3.25	0.604
IGH/BCL2 fusion negative *vs*. positive	69	0.87	0.20–3.67	0.844	0.70	0.22–2.28	0.556
BCL6 break negative *vs*. positive	72	1.50	0.60–3.73	0.387	1.30	0.60–2.82	0.504

Bold font indicates significance. HR, hazard ratio; CI, confidence interval.

# Sample sizes differ due to complete data set per cox model.

**Table 5:** Univariate Cox models for DLBCL, NOS patients treated with R-CHOP.

		OS			PFS		
Characteristic	n#	HR	95% CI	*P*	HR	95% CI	*P*
MYC protein low *vs*. High	118	4.39	2.02–9.54	**<0.001**	2.87	1.66–4.99	**<0.001**
BCL2 protein low *vs*. High	118	1.86	0.92–3.73	0.083	1.47	0.86–2.51	0.157
BCL6 protein low *vs*. High	118	0.33	0.14–0.80	**0.014**	0.71	0.40–1.26	0.238
High Ki67 protein low *vs*. High	118	2.09	1.04–4.23	**0.040**	1.72	1.01–2.95	**0.047**
MYC/BCL2 DHS 0 *vs*. 1 *vs*. 2	118	2.76	1.66–4.60	**<0.001**	2.03	1.39–2.96	**<0.001**
MYC/BCL6 DHS 0 *vs*. 1 *vs*. 2	118	4.23	2.29–7.79	**<0.001**	2.25	1.47–3.45	**<0.001**
BCL2/BCL6 DHS 0 *vs*. 1 *vs*. 2	118	2.11	1.26–3.52	**0.005**	1.37	0.95–1.97	0.089
THS 0 *vs*. 1 *vs*. 2 *vs*. 3	118	2.54	1.70–3.81	**<0.001**	1.72	1.28–2.31	**<0.001**
GCB *vs*. non-GCB	118	1.67	0.69–4.06	0.259	1.97	0.96–4.03	0.064
IPI score of 0–2 *vs.* 3–5	118	1.76	0.79–3.92	0.167	1.74	0.91–3.30	0.093
MYC break negative *vs*. positive	57	0.52	0.07–3.98	0.529	1.19	0.42–3.41	0.740
IGH/BCL2 fusion negative *vs*. positive	55	1.74	0.48–6.36	0.402	0.90	0.32–2.59	0.851
BCL6 break negative *vs*. positive	57	1.04	0.29–3.81	0.948	1.27	0.59–2.73	0.550

Bold font indicates significance.

# Sample sizes differ due to complete data set per cox model.

In the CHOP cohort, multivariate Cox regression model that incorporated IPI, immunohistochemical subgroups (GCB *vs.* non-GCB), MYC, BCL2, and BCL6 proteins showed that IPI, MYC protein, and BCL2 protein maintained independent prognostic values for OS and PFS (*P*<0.05; **Table 6**). Also high MYC/BCL2 DHS, high MYC/BCL6 DHS, high BCL2/BCL6 DHS and high THS maintained independent prognostic values for OS and PFS (all *P*<0.05; **Table 6**).

**Table 6:** Multivariate Cox models for DBCL, NOS patients treated with CHOP (n = 203).

Characteristic	OS			PFS		
	HR	95% CI	*P* *	HR	95% CI	*P* *
IPI score of 0–2 *vs.* 3–5	2.78	1.83–4.23	**<0.001**	2.20	1.52–3.18	**<0.001**
GCB *vs*. non-GCB	1.26	0.74–2.13	0.397	1.49	0.94–2.37	0.092
MYC protein low *vs*. High	1.77	1.14–2.73	**0.010**	1.67	1.15–2.42	**0.007**
BCL2 protein low *vs*. High	1.66	1.05–2.63	**0.032**	1.63	1.10–2.41	**0.015**
BCL6 protein low *vs*. High	0.67	0.42–1.08	0.098	0.66	0.44–0.99	**0.042**
IPI score of 0–2 *vs.* 3–5	2.77	1.82–4.20	**<0.001**	2.20	1.52–3.18	**<0.001**
MYC/BCL2 DHS 0 *vs*. 1 *vs*. 2	1.76	1.30–2.38	**<0.001**	1.68	1.30–2.16	**<0.001**
IPI score of 0–2 *vs.* 3–5	2.86	1.88–4.34	**<0.001**	2.22	1.53–3.21	**<0.001**
MYC/BCL6 DHS 0 *vs*. 1 *vs*. 2	1.73	1.24–2.41	**0.001**	1.66	1.24–2.21	**0.001**
IPI score of 0–2 *vs.* 3–5	2.81	1.84–4.27	**<0.001**	2.14	1.48–3.11	**<0.001**
BCL2/BCL6 DHS 0 *vs*. 1 *vs*. 2	1.63	1.20–2.22	**0.002**	1.68	1.28–2.20	**<0.001**
IPI score of 0–2 *vs.* 3–5	2.79	1.84–4.25	**<0.001**	2.20	1.52–3.18	**<0.001**
THS 0 *vs*. 1 *vs*. 2 *vs*. 3	1.68	1.31–2.16	**<0.001**	1.67	1.34–2.06	**<0.001**

Bold font indicates significance.

*Cox regression enter method.

In the R-CHOP cohort, multivariate Cox regression model that incorporated IPI, MYC, BCL2, and BCL6 proteins showed that only high MYC protein maintained independent prognostic value for both inferior OS (*P*<0.001) and PFS (*P* = 0.001), and low BCL6 protein for inferior OS (*P* = 0.019; **Table 7**). Also high MYC/BCL2 DHS, high MYC/BCL6 DHS, and high THS maintained independent prognostic values for OS and PFS (all *P*<0.05). High BCL2/BCL6 DHS was independent prognostic value or OS (*P* = 0.006; **Table 7**).

**Table 7:** Multivariate Cox models for DBCL, NOS patients treated with R-CHOP (n = 118).

Characteristic	OS			PFS		
	HR	95% CI	*P* *	HR	95% CI	*P* *
IPI score of 0–2 *vs.* 3–5	1.24	0.55–2.80	0.605	1.51	0.78–2.90	0.221
MYC protein low *vs*. High	4.12	1.86–9.10	**<0.001**	2.71	1.55–4.76	**0.001**
BCL2 protein low *vs*. High	1.48	0.71–3.07	0.293	1.35	0.77–2.36	0.303
BCL6 protein low *vs*. High	0.33	0.13–0.83	**0.019**	0.74	0.40–1.37	0.336
Ki67 protein low vs. High	1.71	0.83–3.51	0.144	1.49	0.86–2.57	0.151
IPI score of 0–2 *vs.* 3–5	1.34	0.59–3.03	0.483	1.56	0.81–2.97	0.181
MYC/BCL2 DHS 0 *vs*. 1 *vs*. 2	2.67	1.60–4.48	**<0.001**	1.97	1.35–2.88	**<0.001**
IPI score of 0–2 *vs.* 3–5	1.22	0.54–2.75	0.628	1.44	0.75–2.76	0.270
MYC/BCL6 DHS 0 *vs*. 1 *vs*. 2	4.14	2.23–7.68	**<0.001**	2.19	1.42–3.37	**<0.001**
IPI score of 0–2 *vs.* 3–5	1.50	0.67–3.34	0.326	1.61	0.84–3.07	0.151
BCL2/BCL6 DHS 0 *vs*. 1 *vs*. 2	2.07	1.23–3.50	**0.006**	1.33	0.92–1.94	0.132
IPI score of 0–2 *vs.* 3–5	1.30	0.58–2.91	0.522	1.49	0.78–2.85	0.223
THS 0 *vs*. 1 *vs*. 2 *vs*. 3	2.51	1.67–3.77	**<0.001**	1.69	1.25–2.29	**0.001**

Bold font indicates significance.

*Cox regression enter method.

### Immunohistochemical Subtyping and IPI Predict Survival in DLBCL Patients Treated With CHOP but Not With R-CHOP

**Figure 6:**
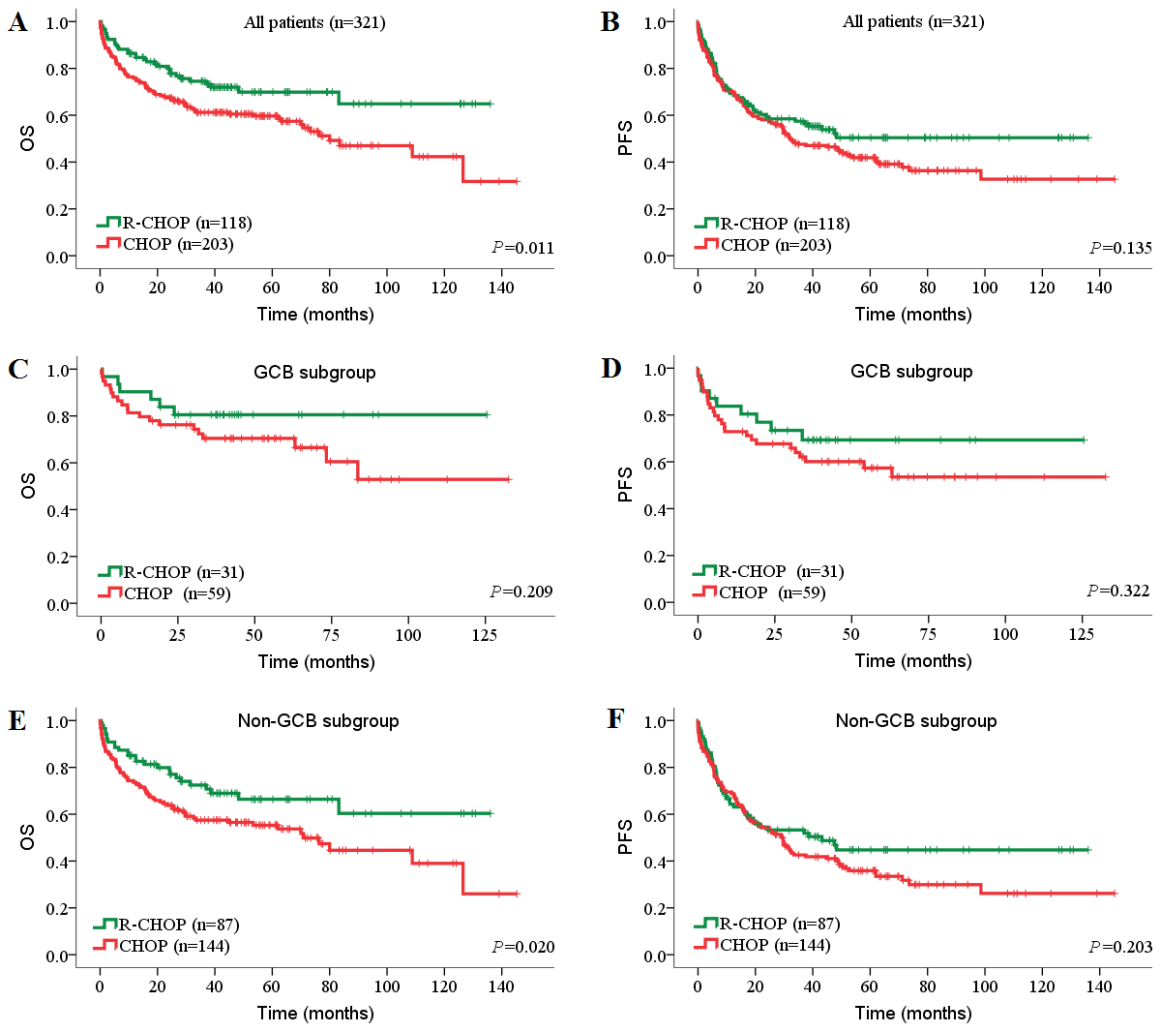
Prognostic impact of treatments in DLBCL risk-stratified according to immunohistochemical subgroups. (A, B) OS (A) and PFS (B) of all patients treated with CHOP or R-CHOP. (C, D) OS (C) and PFS (D) of patients treated with CHOP or R-CHOP in GCB subgroup. (E, F) OS (E) and PFS (F) of patients treated with CHOP or R-CHOP in non-GCB subgroup. OS, overall survival; PFS, progression-free survival; GCB, germinal center B-cell.

For all DLBCL, NOS patients, rituximab significantly improved OS (72.9% *vs*. 56.2%, *P* = 0.011; **Figure 6A**) but not PFS, although a trend was seen (54.2% *vs*. 40.9%, *P* = 0.135; **Figure 6B**). In the GCB subgroup, the OS and PFS showed an increase in the R-CHOP group from survival curves (**Figure 6C–D**), but this was not significant. In the non-GCB subgroup, the patients receiving R-CHOP treatment showed a significantly improved OS than those who received CHOP (70.1% *vs*. 52.1%, *P* = 0.020; **Figure 6E**). These findings were consistent with the results of previous studies in Chinese patients [20], [21]. Importantly, these results indicated that the use of rituximab conferred a clinical benefit to non-GCB-DLBCL patients, frequently associated with poorer prognosis.

**Figure 7:**
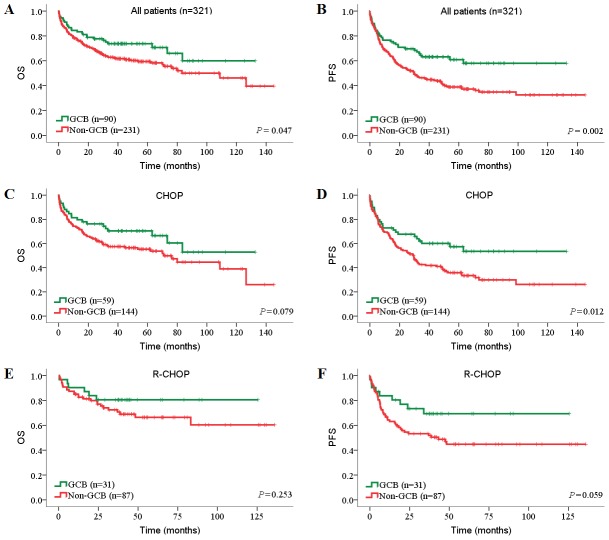
Prognostic impact of immunohistochemical subtypes in DLBCL risk-stratified according to treatments. (A, B) OS (A) and PFS (B) of all patients with GCB subtype or non-GCB subtype. (C, D) OS (C) and PFS (D) of CHOP-treated patients with GCB subtype or non-GCB subtype. (E, F) OS (E) and PFS (F) of R-CHOP-treated patients with GCB subtype or non-GCB subtype. OS, overall survival; PFS, progression-free survival; GCB, germinal center B-cell.

**Figure 8:**
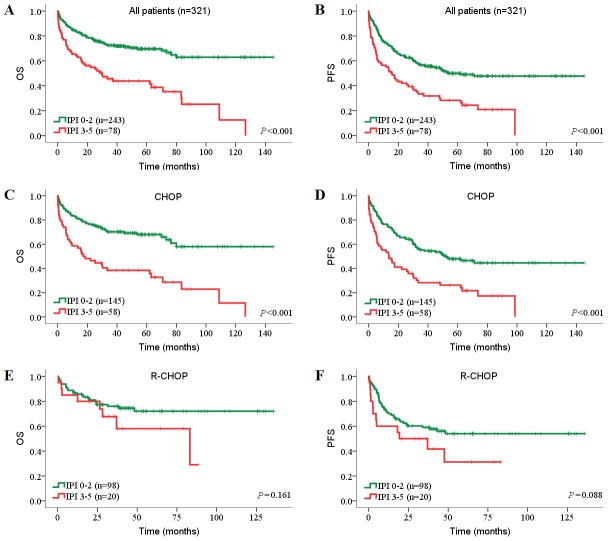
Prognostic impact of IPI in DLBCL risk-stratified according to treatments. (A, B) OS (A) and PFS (B) of all patients with IPI 0–2 or IPI 3–5. (C, D) OS (C) and PFS (D) of CHOP-treated patients with IPI 0–2 or IPI 3–5. (E, F) OS (E) and PFS (F) of R-CHOP-treated patients with IPI 0–2 or IPI 3–5. OS, overall survival; PFS, progression-free survival; IPI, International Prognostic Index.

We further investigated the prognostic value of immunohistochemical subtyping in our series undergoing CHOP or R-CHOP treatment. For all patients, the GCB subgroup had superior OS and PFS than the non-GCB subgroup (OS: 71.1% *vs*. 58.9%, *P* = 0.047; PFS: 62.2% *vs*. 39.4%, *P* = 0.002; **Figure 7A–B**). In the CHOP cohort, the GCB subgroup had superior PFS than the non-GCB subgroup (57.6% *vs*. 34.0%, *P* = 0.012; **Figure 7D**). However, the GCB lost its predictive value in patients treated with rituximab (**Figure 7E–F** **;** **Table 4**). These findings were consistent with the results of previous studies in both Chinese cohorts [19] and Western cohorts [22]. The IPI proved to be highly valuable for both all patients and CHOP-treated patients (**Figure 8A–D**). However, the IPI lost its predictive value in patients who were treated with rituximab which has improved prognosis significantly (**Figure 8E–F**). This result is consistent with previous studies [11], [19] but inconsistent with some other studies [3], [23].

## Discussions

DLBCL, NOS constitutes 25–30% of adult non-Hodgkin lymphomas in Western countries [11] and a higher percentage (37.9%) in Chinese [2]. The poor prognosis of DLBCL patients whose tumors overexpress either MYC or BCL2, or low express BCL6 is well established [24]–[27]. Recently, the negative prognostic impact of coexpression of MYC and BCL2 has been confirmed in DLBCL patients from Western populations treated with R-CHOP [16], [23], [28], [29]. We have confirmed these data in larger series comprising DLBCL, NOS samples from 336 Chinese patients treated with either R-CHOP (n = 125) or CHOP (n = 211). Moreover, we have shown that combined immunohistochemical scores of MYC, BCL2 and BCL6 predict 5-year OS and 5-year PFS in DLBCL patients independent of the IPI following treatment with R-CHOP. Of note, we observed similar results for patients treated with CHOP (data not shown). In contrast, Johnson et al. reported that MYC overexpression predicted poor 3-year OS and event-free survival in R-CHOP-treated patients but not in CHOP-treated patients with unknown mechanism [15]. The contradictory findings may be due to population heterogeneity, different time-to-event end points and different second and/or third line treatments. Different populations treated uniformly within a prospective multicenter trial are needed to further consolidate the prognostic value of MYC combined with BCL2 and BCL6 protein. Importantly, our results indicated that addition of rituximab to standard chemotherapy eliminates the prognostic value of immunohistochemical subgroups (GCB and non-GCB), IPI, BCL2 protein, and BCL2/BCL6 DHS, but enhances the prognostic value of Ki67 in DLBCL.

We here have demonstrated that the analysis of MYC, BCL2 and BCL6 protein expression by IHC is feasible on TMAs in a highly reliable and reproducible manner. High DHSs and THS were associated with many high-risk clinicopathologic features, including high LDH level, poor performance status, advanced stage of disease, multiple extranodal sites of involvement, and high IPI score. Approximately one-third of DLBCL demonstrate MYC/BCL2 DHS 2, in keeping with the 29% and 28% frequency reported by Green et al [16] and Hu et al [29], respectively. By contrast, DHL characterized chromosomal translocations involving *MYC*, *BCL6* and *BCL2* is a rare disease, representing approximately 5% of all DLBCL cases in our study, indicating that, in addition to translocations, protein expression could be regulated by other mechanisms including other types of rearrangements, amplifications, mutation, or by miRNA-depandent mechanisms [30]–[32]. Thus, the findings in this study expand the spectrum of DLBCL, defined at the genetic level, by using IHC.

A number of investigators have attempted to use the immunohistochemical expression patterns as prognostic indicators in DLBCL [33], [34]. Hans et al. reported that a combination of CD10, BCL6 and MUM1 expression could subdivide DLBCL patients into long- and short-term survivors [8]. However, contradictory results have been reported on the prognostic role of the Hans classifier [9], [10], [35]. Although non-GCB subgroup was correlated with inferior 5-year OS and PFS in BLBCL, when stratified by treatment non-GCB was only correlated inferior 5-year PFS in the CHOP cohort. Furthermore, non-GCB was not an independent survival predictor for DLBCL patients treated with CHOP, indicating that such algorithm was not a stable survival predictor for DLBCL especially for R-CHOP-treated patients. Consistent with previous reports [10], [12], [36], [37], Chinese patients with DLBCL in our series had a much lower incidence of GCB subtype compared with those reported on Western populations [23], [37], [38]. These data support the notion that immunophenotypic subgroups in DLBCL vary according to the geographic factor.

Translocations of *MYC*, *BCL2* and *BCL*6 in DLBCL have been consistently reported. Although the prognostic impact of *BCL2* and *BCL6* translocations has been disputed [13], [15], [22], [39], there is consensus that *MYC* translocation is a worse prognostic marker in patients with DLBCL treated CHOP, both in combination with and without rituximab [4], [17], [40]. In our study, univariate and multivariate analyses disclosed that breaks in *MYC* and *BCL6*, as well as fusion in *BCL2* had no impact on survival in CHOP or R-CHOP cohorts.

In our study, *BCL6* breaks and *IGH/BCL2* fusion were observed in 20.0% and 11.1% of Chinese DLBCL cohort, respectively. While in Western cohorts, t(3;14)(q27;q32) involving *BCL6* and t(14;18)(q32;q21) involving IGH and *BCL2* have been found in 20–40% and 20–30%, respectively [15], [41]. Consistent with previous studies [14], [36], [42], the incidence of *BCL-6* break and *IGH/BCL2* fusion were lower in DLBCL in Chinese population compared with Western populations. Similar to what has been previously described [43], we have found a higher frequency of *BCL6* translocations in non-GCB subgroup (25%) than in GCB subgroup (13%). In non-GCB subgroup, BCL6 translocations correlated significantly with high BCL6 expression level. However high BCL6 expression is more common in GCB subgroup, indicating that there may be other molecular mechanisms causing BCL6 over-expression in GCB subgroup. Relocation of an IGH transcriptional enhancer next to the BCL2 gene as a result of the t(14;18) translocation is thought to cause constitutive over-expression of BCL2 protein. In this study, high BCL2 expression showed correlation with *IGH/BCL2* fusion in GCB subgroup but not no correlation with *IGH/BCL2* fusion in GCB subgroup, indicating that BCL2 overexpression in GCB may be due to t(14;18) and in non-GCB is due to other molecular mechanisms. These findings suggest that GCB and non-GCB DLBCL subgroups are two different disease in molecular mechanisms causing the abnormal protein expression. Besides, the low incidence of *IGH/BCL2* fusion in Chinese cohort compared to the Western cohorts may be the possible reason for the lower incidence of GCB-DLBCL in China.

Moreover, our study demonstrate that immunostaining for MYC, BCL2 and BCL6, with the optimal cut off of 90%, 70% and 20%, predict the presence of *MYC* breaks, *IGH/BCL2* fusion and *BCL6* breaks as detected by FISH in DLBCL with high specificity (>90%). Therefore, all patients with protein aberrant expression should be tested for corresponding gene translocation by FISH. Our findings that high MYC and BCL2 protein expression predict gene translocations are consistent with previous studies [31], [36]. However, Akyurek et al. reported that the level of BCL6 protein expression is not correlated with the presence or absence of *BCL6* rearrangement [22]. Different staining and scoring methodologies, cut-off values, and populations may cause this discrepancy. Although *MYC*, *BCL2* and *BCL6* translocations can be detected by FISH, FISH fails to detect gene deregulation caused by mechanisms other than translocation [4]. The availability of monoclonal antibodies that target the MYC, BCL2 and BCL6 protein, respectively, has been shown to predict the corresponding gene rearrangements by our and other independent groups and has been validated for use FFPE tissues [44], allowing for the study of large series of archived DLBCL samples for the protein expressions by IHC.

In conclusion, we show DHL in our series is a rare event and dose not predict survival in Chinese DLBCL, NOS. We confirm that DLBCL with high DHSs or THS characterize a subset of DLBCL patients with high-risk clinicopathologic features and inferior survival. Importantly, we report for the first time that high DHSs and THS are poor prognostic predictors independent of the IPI factor in the Chinese cohort that consisted of R-CHOP-treated patients and CHOP-treated patients. We further show that the incidence of non-GCB subtype in our series is higher than that in the Western populations reported by previous studies, and non-GCB subgroup is not a stable survival predictor for DLBCL, especially for R-CHOP-treated patients. Immunostaining for MYC, BCL2 and BCL6 proved to be an excellent test with high specificity (>90%) for the presence of *MYC* breaks, *IGH/BCL2* fusion and *BCL6* breaks as detected by FISH. Our data together suggest that the combinations of MYC, BCL2 and BCL6 protein expression assessed by IHC are reliable prognostic predictors and could be used in the future as prognostic markers for stratification of patients with DLBCL for novel therapies.

## Supporting Information

Figure S1 **Representative FISH analysis of** ***MYC*** **,** ***BCL2*** **and** ***BCL6*** **rearrangements in diffuse large B-cell lymphoma (DLBCL).** (A) Split signals (orange and green) demonstrating presence of *MYC* break, and (C) *BCL6* break. Fusion signals (orange/green fusion) demonstrating presence of *IGH/BCL2* fusion. (A-C original magnification, ×1000). FISH, fluorescence *in situ* hybridization.
(TIF)

Figure S2 **Prognostic impact of DHL in DLBCL.** (A) OS and (B) PFS of patients with DHL, other patients without DHL (non-DHL), non-DHL with THS 0/1, and non-DHL with THS 2/3. OS, overall survival; PFS, progression-free survival; DHL, double-hit lymphoma; THS, triple-hit score.
(TIF)

Table S1 **Immunohistochemical assays and methods.**
(DOC)

Table S2 **Gene translocation versus immunohistochemical subgroup comparison.**
(DOC)

Table S3 **Patient clinical and immunophenotypical characteristics of patients with DLBCL, NOS based on DHL status or THS.**
(DOC)

Table S4 **Diagnostic performance of protein candidates for gene translocations in DLBCL, NOS based on ROC curves analysis.**
(DOC)

Table S5 **Correlation between MYC protein expression and** ***MYC*** **break in DLBCL, NOS patients.**
(DOC)

Table S6 **Correlation between BCL6 protein expression and** ***BCL6*** **break in DLBCL, NOS patients.**
(DOC)

Table S7 **Correlation between BCL2 protein expression and** ***BCL2*** **break in DLBCL, NOS patients.**
(DOC)
