# Correction: MYC Expression in Concert with BCL2 and BCL6 Expression Predicts Outcome in Chinese Patients with Diffuse Large B-Cell Lymphoma, Not Otherwise Specified

**DOI:** 10.1371/journal.pone.0110724

**Published:** 2014-10-10

**Authors:** The PLOS ONE Staff

The following information is missing from the Funding section: National Clinical Key Subject Construction Project Fund of China funded this work. This funder had no role in study design, data collection and analysis, decision to publish, or preparation of the manuscript.
