# Correction: MYC Expression in Concert With BCL2 and BCL6 Expression Predicts Outcome in Chinese Patients With Diffuse Large B-Cell Lymphoma, Not Otherwise Specified

**DOI:** 10.1371/journal.pone.0210027

**Published:** 2018-12-26

**Authors:** Li-Xu Yan, Yan-Hui Liu, Dong-Lan Luo, Fen Zhang, Yu Cheng, Xin-Lan Luo, Jie Xu, Jie Cheng, Heng-Guo Zhuang

There is an error in affiliation 1. Affiliation 1 should be: Department of Pathology, Guangdong General Hospital, Guangdong Academy of Medical Sciences, Guangzhou, Guangdong.
