# Supplementary figures and images for: MYC Expression in Concert with BCL2 and BCL6 Expression Predicts Outcome in Chinese Patients with Diffuse Large B-Cell Lymphoma, Not Otherwise Specified
Source: PLoS One. 2014 Aug 4;9(8):e104068. doi: 10.1371/journal.pone.0104068 (PMC4121314; doi:10.1371/journal.pone.0104068)

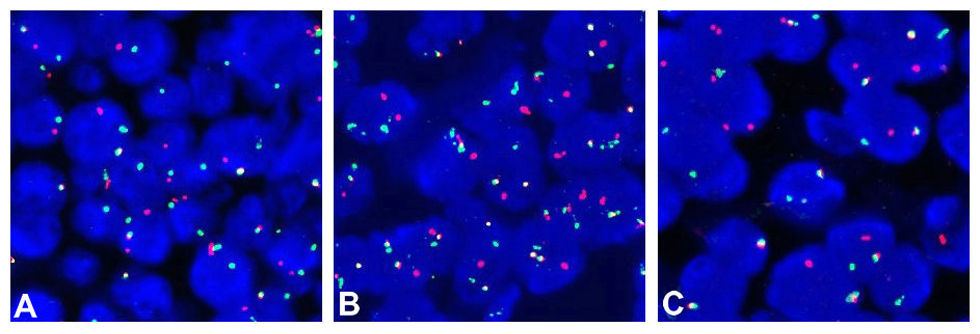

Supplement: Figure S1 — Representative FISH analysis of MYC , BCL2 and BCL6 rearrangements in diffuse large B-cell lymphoma (DLBCL). (A) Split signals (orange and green) demonstrating presence of MYC break, and (C) BCL6 break. Fusion signals (orange/green fusion) demonstrating presence of IGH/BCL2 fusion. (A-C original magnification, ×1000). FISH, fluorescence in situ hybridization. (TIF) [file pone.0104068.s001.tif]

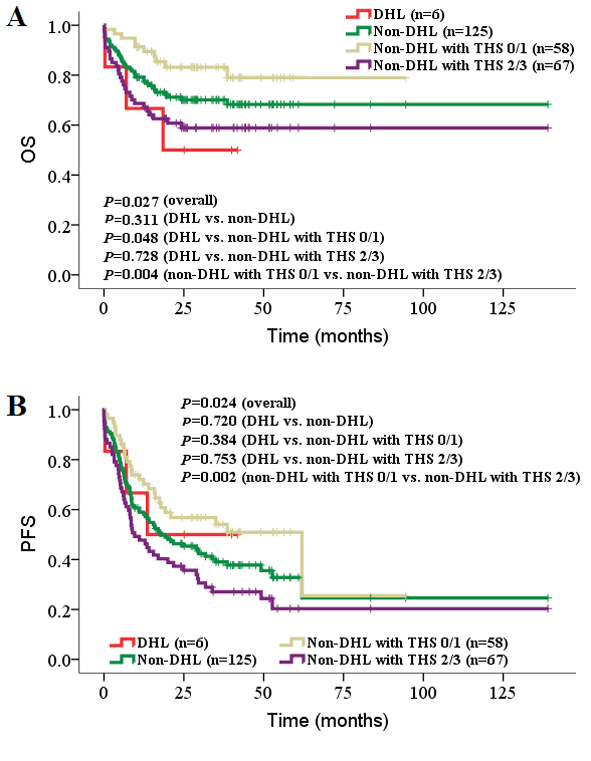

Supplement: Figure S2 — Prognostic impact of DHL in DLBCL. (A) OS and (B) PFS of patients with DHL, other patients without DHL (non-DHL), non-DHL with THS 0/1, and non-DHL with THS 2/3. OS, overall survival; PFS, progression-free survival; DHL, double-hit lymphoma; THS, triple-hit score. (TIF) [file pone.0104068.s002.tif]
