# Supplementary material for: MYC Expression in Concert with BCL2 and BCL6 Expression Predicts Outcome in Chinese Patients with Diffuse Large B-Cell Lymphoma, Not Otherwise Specified
Source: PLoS One. 2014 Aug 4;9(8):e104068. doi: 10.1371/journal.pone.0104068 (PMC4121314; doi:10.1371/journal.pone.0104068)
Supplement: Table S1 — Immunohistochemical assays and methods. (DOC) [file pone.0104068.s003.doc]

**Table S1.** Immunohistochemical assays and methods.

| **Antibody** | **Clone** | **Source** | **Retrieval** | **Dilution** |
| --- | --- | --- | --- | --- |
| MYC | Y69 | Epitomics, Burlingame, CA | Mild CC1 | 1:100 |
| BCL2 | 124 | DAKO, Glostrup, Denmark | ER2, 25minutes | 1:200 |
| BCL6 | PG-B6p | DAKO, Glostrup, Denmark | ER2, 25minutes | 1:100 |
| MUM1 | MUM1p | DAKO, Glostrup, Denmark | ER2, 25minutes | 1:1600 |
| CD10 | 56C6 | Leica Microsystems, Ballerup, Denmark | ER2, 25minutes | 1:100 |
| Ki67 | MIB1 | DAKO, Glostrup, Denmark | ER2, 25minutes | 1:100 |
| CD138 | MI15 | DAKO, Glostrup, Denmark | Mild CC1 | 1:1600 |
| CD30 | Ber-H2 | DAKO, Glostrup, Denmark | ER2, 25minutes | 1:400 |
| CD5 | SP19 | ZETA, CA, USA | ER2, 25minutes | 1:100 |
| ALK | ALK1 | DAKO, Glostrup, Denmark | ER2, 25minutes | 1:100 |
| CD3 | Code:IS503 | DAKO, Glostrup, Denmark | ER2, 25minutes | 1:200 |
| CD20 | L26 | DAKO, Glostrup, Denmark | Mild CC1 | 1:800 |
| CD79α | JCB117 | DAKO, Glostrup, Denmark | Mild CC1 | 1:200 |
| CD45RO | UCHL1 | DAKO, Glostrup, Denmark | Mild CC1 | 1:100 |
| Cyclin D1 | EP12 | DAKO, Glostrup, Denmark | Mild CC1 | 1:100 |
| CD21 | 1F8 | DAKO, Glostrup, Denmark | ER2, 25minutes | 1:200 |
| CD23 | DAK-CD23 | DAKO, Glostrup, Denmark | ER2, 25minutes | 1:200 |
| PAX-5 | DAK-Pax5 | DAKO, Glostrup, Denmark | ER2, 25minutes | 1:150 |
| OCT-2 | sc-56822 | Santa Cruz, Texas, U.S.A | Mild CC1 | 1:500 |

Abbreviations: CC1, Cell Conditioner 1; Leica: Bond-Max.
