# Supplementary material for: MYC Expression in Concert with BCL2 and BCL6 Expression Predicts Outcome in Chinese Patients with Diffuse Large B-Cell Lymphoma, Not Otherwise Specified
Source: PLoS One. 2014 Aug 4;9(8):e104068. doi: 10.1371/journal.pone.0104068 (PMC4121314; doi:10.1371/journal.pone.0104068)
Supplement: Table S2 — Gene translocation versus immunohistochemical subgroup comparison. (DOC) [file pone.0104068.s004.doc]

**Table S2. Gene translocation versus immunohistochemical subgroup comparison.**

| **Gene rearrangement** | **n/Total N (%)** | **GCB** | **Non-GCB** | ***P*** |
| --- | --- | --- | --- | --- |
| MYC break | 14/133 (11) | 7/46 (15) | 7/87 (8) | 0.239^*^ |
| IGH/BCL2 fusion | 14/124 (11) | 7/43 (16) | 7/81 (9) | 0.238^#^ |
| BCL6 break | 27/129 (21) | 6/46 (13) | 21/83 (25) | 0.101^*^ |

NOTE. Data are given as number/total number (%).

Abbreviations: GCB, germinal center B-cell; non-GCB, non-germinal center B-cell. ^*^Fisher's exact test. ^#^Pearson's Chi-Square test.
