# Supplementary material for: MYC Expression in Concert with BCL2 and BCL6 Expression Predicts Outcome in Chinese Patients with Diffuse Large B-Cell Lymphoma, Not Otherwise Specified
Source: PLoS One. 2014 Aug 4;9(8):e104068. doi: 10.1371/journal.pone.0104068 (PMC4121314; doi:10.1371/journal.pone.0104068)
Supplement: Table S3 — Patient clinical and immunophenotypical characteristics of patients with DLBCL, NOS based on DHL status or THS. (DOC) [file pone.0104068.s005.doc]

**Table S3. Patient clinical and immunophenotypical characteristics of patients with DLBCL, N**OS based on DHL status or THS.

|  | **All patients** | **DHL** | **Non-DHL** |  | **Non-DHL with THS 2/3** |  | **Non-DHL with THS 0/1** |  |
| --- | --- | --- | --- | --- | --- | --- | --- | --- |
| **Characteristic** | **(n=131)** | **(n=6)** | **(n=125)** | ***P* (DHL vs. non-DHL)** | **(n=67)** | ***P* (DHL vs. non-DHL with THS 2/3)** | **(n=58)** | ***P* (DHL vs. non-DHL with THS 0/1)** |
| LDH > ULN | 51 (39) | 3 (50) | 48 (38) | 0.888^*^ | 29 (43) | 1.000^*^ | 19 (33) | 0.693^*^ |
| ECOG PS ≥ 2 | 24 (18) | 3 (50) | 21 (17) | 0.130^*^ | 12 (18) | 0.181^*^ | 9 (16) | 0.131^*^ |
| Stage III/IV | 56 (43) | 3 (50) | 53 (42) | 1.000^*^ | 30 (45) | 1.000^*^ | 23 (40) | 0.956^*^ |
| E sites ≥2 | 16 (12) | 1 (17) | 15 (11) | 0.550^#^ | 6 (9) | 0.466^#^ | 9 (16) | 1.000^#^ |
| IPI score of 3-5 | 27 (21) | 2 (33) | 25 (20) | 0.786^*^ | 16 (24) | 0.984^*^ | 9 (16) | 0.594^*^ |
| Immunohistochemical subgroups |  |  |  |  |  |  |  |  |
| CD5-positive | 11 (8) | 0 (0) | 11 (9) |  | 9 (13) |  | 2 (3) |  |
| GCB | 43 (33) | 2 (33) | 41 (33) | 1.000^*^ | 14 (21) | 1.000^*^ | 27 (47) | 0.792^*^ |
| non-GCB | 77 (59) | 4 (67) | 73 (58) |  | 44 (66) |  | 29 (50) |  |
| High MYC expression | 79 (60) | 4 (67) | 75 (60) | 1.000^*^ | 57 (85) | 0.254^#^ | 18 (31) | 0.194^*^ |
| High BCL2 expression | 76 (58) | 5 (83) | 71 (57) | 0.388^*^ | 59 (88) | 0.560^#^ | 12 (21) | **0.005**^*^ |
| High BCL6 expression | 57 (44) | 3 (50) | 54 (43) | 1.000^*^ | 13 (19) | 0.222^*^ | 41 (71) | 0.563^*^ |
| High Ki67 expression | 83 (63) | 3 (50) | 80 (64) | 0.794^*^ | 43 (64) | 0.804^*^ | 37 (64) | 0.825^*^ |

NOTE. Data are given as number (%). Bold font indicates significance.

Abbreviations: DLBCL, diffuse large B-cell lymphoma; NOS, not otherwise specified; DHL, double-hit lymphoma; THS, triple-hit score; LDH, lactate dehydrogenase; ULN, upper limit of normal; ECOG PS, Eastern Cooperative Oncology Group performance status; E sites, extranodal sites; IPI, international prognostic index; GCB, germinal center B-cell; non-GCB, non-germinal center B-cell.

^*^Correction for continuity. ^#^Fisher's exact test.
