# Supplementary material for: MYC Expression in Concert with BCL2 and BCL6 Expression Predicts Outcome in Chinese Patients with Diffuse Large B-Cell Lymphoma, Not Otherwise Specified
Source: PLoS One. 2014 Aug 4;9(8):e104068. doi: 10.1371/journal.pone.0104068 (PMC4121314; doi:10.1371/journal.pone.0104068)
Supplement: Table S4 — Diagnostic performance of protein candidates for gene translocations in DLBCL, NOS based on ROC curves analysis. (DOC) [file pone.0104068.s006.doc]

**Table S~~4~~. Diagnostic performance of protein candidates for gene translocations in DLBCL, NOS** based on ROC curves analysis.

|  | **Cut-off values** | **Sensitivity** | **Specificity** | **AUC (95% confidence interval)** | **LR+**  **(+LR)** | **PPV**  **(-LR)** | ***P*** |
| --- | --- | --- | --- | --- | --- | --- | --- |
| MYC protein | ≥90% | 50.0 | 95.3 | 0.73 (0.65-0.80) | 10.7 | 0.52 | 0.001 |
| BCL2 protein | ≥70% | 15.4 | 94.7 | 0.55 (0.46-0.64) | 2.92 | 0.89 | 0.046 |
| BCL6 protein | ≥20% | 26.0 | 93.2 | 0.60 (0.51-0.68) | 3.82 | 0.79 | 0.001 |

Abbreviations: DLBCL, diffuse large B-cell lymphoma; ROC, Receiver Operating Characteristic; AUC, area under the ROC curve; LR, likelihood ratio; PPV, positive predictive value.
