# Supplementary material for: MYC Expression in Concert with BCL2 and BCL6 Expression Predicts Outcome in Chinese Patients with Diffuse Large B-Cell Lymphoma, Not Otherwise Specified
Source: PLoS One. 2014 Aug 4;9(8):e104068. doi: 10.1371/journal.pone.0104068 (PMC4121314; doi:10.1371/journal.pone.0104068)
Supplement: Table S5 — Correlation between MYC protein expression and MYC break in DLBCL, NOS patients. (DOC) [file pone.0104068.s007.doc]

**Table S5. Correlation between MYC protein expression and *MYC* break in DLBCL, NOS patients.**

|  | ***MYC* break** | |  |
| --- | --- | --- | --- |
|  | **Positive** | **Negative** | ***P*** |
| **DLBCL, NOS, n=144** |  |  |  |
| MYC low (<90%) | 6/128 (5) | 122/128 (95) |  |
| MYC high (≥90%) | 8/16 (50) | 8/16 (50) | <0.001^*^ |
| **GCB subgroup, n=46** |  |  |  |
| MYC low (<90%) | 4/43 (9) | 39/43 (91) |  |
| MYC high (≥90%) | 3/3 (100) | 0/3 (0) | 0.002^#^ |
| **Non-GCB subgroup, n=87** |  |  |  |
| MYC low (<90%) | 2/77 (3) | 75/77 (97) |  |
| MYC high (≥90%) | 5/10 (50) | 5/10 (50) | <0.001^#^ |

NOTE. Data are given as number/total number (%).

Abbreviations: DLBCL, diffuse large B-cell lymphoma. ^*^Correction for continuity. ^#^Fisher's exact test.
