# Supplementary material for: MYC Expression in Concert with BCL2 and BCL6 Expression Predicts Outcome in Chinese Patients with Diffuse Large B-Cell Lymphoma, Not Otherwise Specified
Source: PLoS One. 2014 Aug 4;9(8):e104068. doi: 10.1371/journal.pone.0104068 (PMC4121314; doi:10.1371/journal.pone.0104068)
Supplement: Table S6 — Correlation between BCL6 protein expression and BCL6 break in DLBCL, NOS patients. (DOC) [file pone.0104068.s008.doc]

**Table S6. Correlation between BCL6 protein expression and *BCL6* break in DLBCL, NOS patients.**

|  | ***BCL6* break** | |  |
| --- | --- | --- | --- |
|  | **Positive (%)** | **Negative (%)** | ***P*** |
| **DLBCL, NOS, n=140** |  |  |  |
| BCL6 low (<20%) | 3 (7) | 41 (93) |  |
| BCL6 high (≥20%) | 25 (26) | 71 (74) | 0.008^#^ |
| **GCB subgroup, n=46** |  |  |  |
| BCL6 low (<20%) | 0/9 (0) | 9/9 (100) |  |
| BCL6 high (≥20%) | 6/37 (16) | 31/37 (84) | 0.457^*^ |
| **Non-GCB subgroup, n=83** |  |  |  |
| BCL6 low (<20%) | 3/31 (10) | 28/31 (90) |  |
| BCL6 high (≥20%) | 18/52 (35) | 34/52 (65) | 0.011^#^ |

NOTE. Data are given as number/total number (%).

Abbreviations: DLBCL, diffuse large B-cell lymphoma. ^#^Pearson's Chi-Square test. ^*^Correction for continuity.
