# Supplementary material for: MYC Expression in Concert with BCL2 and BCL6 Expression Predicts Outcome in Chinese Patients with Diffuse Large B-Cell Lymphoma, Not Otherwise Specified
Source: PLoS One. 2014 Aug 4;9(8):e104068. doi: 10.1371/journal.pone.0104068 (PMC4121314; doi:10.1371/journal.pone.0104068)
Supplement: Table S7 — Correlation between BCL2 protein expression and BCL2 break in DLBCL, NOS patients. (DOC) [file pone.0104068.s009.doc]

**Table S7. Correlation between BCL2 protein expression and *BCL2* break in DLBCL, NOS patients.**

|  | ***IGH/BCL2* fusion** | |  |
| --- | --- | --- | --- |
|  | **Positive** | **Negative** | ***P*** |
| **DLBCL, NOS, n=135** |  |  |  |
| BCL2 low (<70%) | 3/57 (5) | 54/57 (95) |  |
| BCL2 high (≥70%) | 12/78 (15) | 66/78 (85) | 0.065^#^ |
| **GCB subgroup, n=43** |  |  |  |
| BCL2 low (<70%) | 1/24 (4) | 23/24 (96) |  |
| BCL2 high (≥70%) | 6/19 (32) | 13/19 (68) | 0.045^*^ |
| **Non-GCB subgroup, n=81** |  |  |  |
| BCL2 low (<70%) | 2/29 (7) | 27/29 (93) |  |
| BCL2 high (≥70%) | 5/52 (10) | 47/52 (90) | 0.996^*^ |

NOTE. Data are given as number/total number (%).

Abbreviations: DLBCL, diffuse large B-cell lymphoma. ^#^Pearson's Chi-Square test. ^*^Correction for continuity.
